# Supplementary material for: The effect of mental health on sleep quality of front-line medical staff during the COVID-19 outbreak in China: A cross-sectional study
Source: PLoS One. 2021 Jun 24;16(6):e0253753. doi: 10.1371/journal.pone.0253753 (PMC8224907; doi:10.1371/journal.pone.0253753)
Supplement: Attachment — Submitted filename: 1.Response to Reviewers.docx [file pone.0253753.s002.docx]

**Dear editor:**

Thanks a lot for your reviews to our manuscript. We acknowledge your comments and constructive suggestions very much, which are valuable for improving the quality of our manuscript. We have revised the manuscript according to the reviewers’ comments in detail. We hope, with these modifications and improvements based on your suggestions and the reviewers’ comments, the quality of our manuscript would meet the publication standard of PLOS ONE.

The revisions have been done in the attached manuscript. Some explanations regarding the revisions of our manuscript are as follows. If you have any question, please contact us without hesitate.

Thank you and best regards.

Yours sincerely,

Xiandong Meng

Address: #37 Guoxue Alley, Wuhou District, Chengdu, Sichuan Province, P.R. China

Phone: 028-85422628

E-mail: wchmhcmxd@163.com

**Reply to the reviewers’ comments**

| **Reviewer** | **Number** | **Original comments of the reviewer** | **Reply by the author(s)** | **Changes done on page number and line number** |
| --- | --- | --- | --- | --- |
| **Academic editor** | **Q1** | Please ensure that your manuscript meets PLOS ONE's style requirements, including those for file naming. | Thank you for bringing up this important point. We have revised the format of the article as required | **Please see the full text.** |
|  | **Q2** | In your Methods section, please provide additional information about the participant recruitment method and the demographic details of your participants. Please ensure you have provided sufficient details to replicate the analyses such as:   1. a description of any inclusion/exclusion criteria that were applied to participant recruitment. 2. a statement as to whether your sample can be considered representative of a larger population.      3. a description of how participants were recruited. 4. descriptions of where participants were recruited and where the research took place. 5. a sample size calculation. | Thank you for bringing up this important point. We have revised the Methods section and supplemented the content which was omitted before.   1. This study included 543 FMS from a medical center in Western China who regularly treated or were in contact with patients infected with COVID-19, during a period spanning from February, 2020 to March, 2020. The inclusion criteria were as follows: (1) regular employees, (2) worked at their posts during the survey, (3) WeChat users. All study participants willingly volunteered to participate in the study. 2. The subjects of this study were selected from the medical staff of West China Hospital of Sichuan University，which is one of the largest medical centers in China. After the outbreak of the epidemic, we not only sent medical staff to Wuhan to treat the infected people in the first time, but also undertook the treatment of the infected people in the area where the hospital is located. Besides, in this study, 84.4% of the research population were nurses, and 94.3% of the research population were women, which was consistent with the occupation and sex distribution of the Chinese healthcare system. However, our survey subjects were derived from one specific hospital through convenience sampling, thus the representativeness may be reduced, which may be treated as a limitation of the study. 3. We contacted department heads in each department and invited them to forward our questionnaire to their WeChat group of staff to recruit participants. 4. The questionnaire was built on a professional questionnaire survey network platform called“Wenjuan Xing” (www.wjx.cn) and then was shared on social media WeChat. 5. Sample size was determined using the formula:   $N=\frac{z^{2}\times p\left( 1-p \right)}{e^{2}}$  where ‘z’ is 1.96 at 95% confidence interval, ‘e’ is margin of error at 5% and ‘p’ is prevalence rate of 40% from a recent study done in China. | **Page 6 and 7, line 90-114** |
|  | **Q3** | About financial disclosure: please address the following queries:   a. Please clarify the sources of funding (financial or material support) for your study. List the grants or organizations that supported your study, including funding received from your institution. b. State what role the funders took in the study. If the funders had no role in your study, please state: “The funders had no role in study design, data collection and analysis, decision to publish, or preparation of the manuscript.” c. If any authors received a salary from any of your funders, please state which authors and which funders. | a. This research was received grant from funding 2019 novel coronavirus disease technology research project of West China Hospital of Sichuan University (NO: HX-2019-nCov-034). b. The funders had no role in study design, data collection and analysis, decision to publish, or preparation of the manuscript. c. None of the authors received a salary from the funders. |  |
|  | **Q4** | Please ensure that you have an ORCID iD and that it is validated in Editorial Manager. | ORCID iD:0000-0003-3270-9666 |  |
| **Reviewer #1** | **Abstract** | Kindly improve the presentation of the structured abstract. In the presentation of results, it is suggested to use only P-values instead of OR, CI. | Thanks for the detailed suggestion. We have modified it according to your suggestion. | **Page 2 and 3, line16-25** |
|  | **Introduction** | It is suggested to include the data of the outbreak during the period when the study was conducted to provide more context to interpret the findings. | Thanks for the detailed suggestion. We supplemented studies on the incidence of anxiety, depression, and sleep disorders among health care workers during the epidemic. | **Page 3 and 4, line53-63** |
|  | **Methods** | 1. Is the study conducted physically or by using an online platform to administer the questionnaire? 2. Is there any strategy used to address the missing data? 3. There is no description, how the sample size was calculated, and how the participants were recruited to achieve the final number of participants for analysis? 4. There are no details provided on what basis multivariate logistic regression applied to different variables. | Thank you for your insightful advisement and comments on the Methods section. All your questions are answered below.   1. The questionnaire was built on a professional questionnaire survey network platform called “Wenjuan Xing” (www.wjx.cn) and then was shared on social media WeChat. 2. While constructing the online questionnaire, the integrity check function of the platform was used, meaning the questionnaire could not be submitted unless all questions were answered. 3. Sample size was determined using the formula:   $N=\frac{z^{2}\times p\left( 1-p \right)}{e^{2}}$  where ‘z’ is 1.96 at 95% confidence interval, ‘e’ is margin of error at 5% and ‘p’ is prevalence rate of 40% from a recent study done in China. According to the formula, N=369, considering the non-response rate of 20%, at least 443 sample size are needed. We contacted department heads in each department and invited them to forward our questionnaire to their WeChat group of staff to recruit participants. A total of 546 FMS completed the questionnaire survey, which included three participants were disagreeing to use their questionnaire for study due to their answers are worthless(n=2), worried about expose their privacy(n=1). Hence,543 effective questionnaires for analysis.   4. Multivariate logistic regression analysis was performed on the variables that were significant in univariate analysis. | **Page 6 and 7, line 90-114** |
|  | **Results** | 1. It is advisable to adopt a dual approach to report cut-offs of BDI, SAS, and PSQI with its continuous score in table 1. 2. Kindly clarify what test applied to analyze the factors affecting sleep quality is it the Chi-square test or logistic regression analysis also revise p-values presented in table 2. | 1. Thanks for the detailed suggestion. We have made modifications according to your suggestions and the format of the whole table has been revised. 2. The binary logistic regression analyses were used to estimate the odds ratio for each independent variable, to assess which of the factors associated with poor sleep quality. In addition, p-values presented in table 2 have been revised. | **Page 10 and 11, Table 1.**  **Page 12 and 13, Table 2.** |
|  | **Discussion** | To improve the discussion section and as well as the introduction section it is suggested to provide a link between the effect of COVID-19 on the mental health & sleep quality of healthcare workers. | Thank you for pointing it out. In fact, anxiety and depression have bidirectional association with sleep quality. Particularly during the Covid-19 outbreak, FMS faced greater risk of infection and work stress, as well as frequent policy changes, unclear case management criteria, and other ambiguous conditions that led to depression and anxiety, which have contributed to the higher incidence of sleep disorders. | **Page 22, line 309-326.** |
|  | **Limitation** | Is there any strategy used to remove biases, if not mention it as a limitation to the current study. | Thank you for bringing up this important point. The participants completed the self-reported questionnaires using the WeChat application and mobile devices, which might lead to self-selection bias. Therefore, it will be a limitation of this study. | **Page 24, line 361-372.** |
| **Reviewer #2** |  | In my opinion, introduction, results and discussion are properly reported. My only concern refers to the sample size. Authors should explain whether their sample was collected by convenience or a preventive power analysis was otherwise modelled. It is particularly important as Authors did perform a multivariate analysis, whose reliability should be discussed accordingly to the actual reliability of statistical estimates. | Thank you very much for your very important suggestions and comments on the sample size. In fact, a convenience sampling method was used to recruit participants in this study. According to inclusion and exclusion criteria，a total of 546 FMS completed the questionnaire survey, which included three participants were disagreeing to use their questionnaire for study due to their answers are worthless(n=2), worried about expose their privacy(n=1), 543 effective questionnaires for analysis. Based on the calculation method of cross-sectional study sample size, at least 443 sample size are needed for this study. Hence, the participants we recruited reached the estimated sample size.  Sample size was determined using the formula:  $N=\frac{z^{2}\times p\left( 1-p \right)}{e^{2}}$  where ‘$z$’ is 1.96 at 95% confidence interval, ‘$e$’ is margin of error at 5% and ‘$p$’is prevalence rate of 40% from a recent study done in China. According to the formula, N=369, considering the non-response rate of 20%, at least 443 sample size are needed. | **Page 6, line 108-114.** |
