# Supplementary material for: The effect of mental health on sleep quality of front-line medical staff during the COVID-19 outbreak in China: A cross-sectional study
Source: PLoS One. 2021 Jun 24;16(6):e0253753. doi: 10.1371/journal.pone.0253753 (PMC8224907; doi:10.1371/journal.pone.0253753)
Supplement: Attachment — Submitted filename: 2.Response to Reviewers.docx [file pone.0253753.s003.docx]

**Dear editor:**

Thanks a lot for your reviews to our manuscript. We acknowledge your comments and constructive suggestions very much, which are valuable for improving the quality of our manuscript. We have revised the manuscript according to the reviewers’ comments in detail. We hope, with these modifications and improvements based on your suggestions and the reviewers’ comments, the quality of our manuscript would meet the publication standard of PLOS ONE.

The revisions have been done in the attached manuscript. Some explanations regarding the revisions of our manuscript are as follows. If you have any question, please contact us without hesitate.

Thank you and best regards.

Yours sincerely,

Xiandong Meng

Address: #37 Guoxue Alley, Wuhou District, Chengdu, Sichuan Province, P.R. China

Phone: 028-85422628

E-mail: wchmhcmxd@163.com

**Reply to the reviewers’ comments**

| **Reviewer** | **Number** | **Original comments of the reviewer** | **Reply by the author(s)** |
| --- | --- | --- | --- |
| **Academic editor** | **Q1** | Please review your reference list to ensure that it is complete and correct. | Thank you for bringing up this important point. We have revised the format of the references as required. In addition, two references were replaced, the 33rd and 35th respectively. The 33rd is the research published in Chinese journals, and the 35th is the operation manual. Readers may not get the full text, so we use the relevant references instead. |
| **Reviewer #2** | **Q1** | Sample size was determined using the formula:  $N=\frac{z^{2}\times p\left( 1-p \right)}{e^{2}}$  where ‘z’ is 1.96 at 95% confidence interval, ‘e’ is margin of error at 5% and ‘p’ is  prevalence rate of 40% from a recent study done in China. According to the formula, N=369,  It is rather unclear what the prevalence rate of 0.4 refers to. Please explain. | Thank you for pointing it out. In fact, the aim of this study was to investigate the sleep quality of FMS during the COVID-19 outbreak in China and analyze the relationship between mental health and sleep quality of FMS. The logistic regression analyses were used to estimate the odds ratio for each independent variable, to assess which of the factors associated with sleep disorders. Therefore, the prevalence rate of 0.4 refers to the prevalence rate of sleep disorders in Chinese healthcare professionals under the outbreak of COVID-19. |
